# Dietary fatty acid intake and gut microbiota determine circulating endocannabinoidome signaling beyond the effect of body fat

**DOI:** 10.1038/s41598-020-72861-3

**Published:** 2020-09-29

**Authors:** Sophie Castonguay-Paradis, Sébastien Lacroix, Gabrielle Rochefort, Lydiane Parent, Julie Perron, Cyril Martin, Benoît Lamarche, Frédéric Raymond, Nicolas Flamand, Vincenzo Di Marzo, Alain Veilleux

**Affiliations:** 1 Centre Nutrition, Santé et Société (NUTRISS), Institut sur la nutrition et les aliments fonctionnels (INAF), Université Laval, 2440, boulevard Hochelaga, Québec, QC G1V 0A6 Canada; 2 Centre de recherche de l’Institut universitaire de cardiologie et de pneumologie de Québec (IUCPQ), Université Laval, Québec, QC Canada; 3 École de nutrition, Faculté des sciences de l’agriculture et de l’alimentation (FSAA), Université Laval, Québec, QC Canada; 4 Département de médecine, Faculté de Médecine, Université Laval, Québec, QC Canada; 5 Joint International Unit on Chemical and Biomolecular Research on the Microbiome and Its Impact on Metabolic Health and Nutrition (UMI-MicroMeNu), Pozzuoli, Italy; 6 Canada Research Excellence Chair in the Microbiome-Endocannabinoidome Axis in Metabolic Health (CERC-MEND), Université Laval, Québec, QC Canada

**Keywords:** Metabolism, Microbiota, Obesity

## Abstract

The endocannabinoidome encompasses several fatty acid (FA)-derived mediators, including the endocannabinoid anandamide (AEA) and 2-arachidonoyl-glycerol (2-AG), which served as targets for anti-obesity drug development, and their congener *N*-acyl-ethanolamines (NAEs) and 2-monoacyl-glycerols (2‑MAGs), which are involved in food intake and energy metabolism. Body weight and fat distribution have been suggested as determinants of peripheral endocannabinoid levels. We aimed at investigating factors, beyond body fat composition, that are associated with circulating NAE and 2-MAG levels in a heterogeneous human population. Plasma NAEs and 2-MAGs were measured using LC–MS/MS in a cross-sectional sample of healthy men and women (n = 195) covering a wide range of BMI and individuals before and after a 2-day Mediterranean diet (n = 21). Circulating levels of all 2-MAGs and NAEs, other than *N*-oleoyl-ethanolamine (OEA), correlated with body fat mass and visceral adipose tissue (0.26 < r < 0.54). NAE levels were elevated in individuals with elevated fat mass, while 2-MAGs were increased in individuals with predominantly visceral body fat distribution. Dietary intakes of specific FAs were associated with 2-AG and omega-3-FA-derived NAEs or 2-MAGs, irrespective of the body fat distribution. Some gut bacterial families (e.g. *Veillonellaceae*, *Peptostreptococcaceae* and *Akkermansiaceae*) were associated with variations in most NAEs or omega-3-FA-derived 2‑MAGs, independently of fat mass and dietary FA intake. Finally, a 2-day Mediterranean diet intervention increased circulating levels of NAEs and 2-MAGs in agreement with changes in FA intake (p < 0.01). Self-reported intake and short-term dietary intervention increased in oleic acid and EPA and DHA intake as well as certain gut microbiota taxa are associated to circulating NAEs and 2‑MAGs independently of adiposity measures, thus highlighting the potential importance of these variables in determining endocannabinoidome signaling in humans.

## Introduction

Endocannabinoids are long chain omega-6 polyunsaturated fatty acid (PUFA)-derived lipid mediators involved in energy metabolism in brain and peripheral tissues^1^. The two endocannabinoids, *N*-arachidonoyl-ethanolamine (AEA) and 2-arachidonoyl-glycerol (2-AG), are endogenous agonists of the cannabinoid receptors CB_1_ and CB_2_^2^. *N*-acyl-ethanolamines (NAEs) and 2-monoacyl-glycerols (2‑MAGs), the congeners of AEA and 2-AG, respectively, do not activate the CB_1_ and CB_2_ receptors efficiently. Instead, these congeners activate other receptors (e.g. peroxisome proliferator-activated receptors (PPAR)-α/γ, G-protein coupled receptors (GPR) 55, 110, 119, TRPV1 channels), which, unlike CB_1_, are known to counteract metabolic disorders in animal models^3–5^. These congeners along with AEA and 2-AG, however, have the property of being mostly produced an inactivated by the same enzymes^2^. All these biomolecular entities, together with other bioactive long chain fatty acid amides, their receptors and metabolic enzymes, form a major extension of the endocannabinoid system, known as the endocannabinoidome^2^.

NAEs and 2-MAGs interact with different receptors, distributed in several tissues and exerting pleiotropic and sometimes opposing metabolic effects. One of the most accepted roles of endocannabinoids is the regulation of energy metabolism through CB_1_, in particular by increasing food intake, inhibiting energy expenditure and favouring fat accumulation in the adipose tissue^1,6,7^. Indeed, CB_1_ antagonists have been developed for the treatment of obesity and its consequences^8–10^, and the levels of the main endocannabinoid were linked with adiposity in several studies^11–18^.

A limited number of studies documented a relationship between dietary lipid intakes and circulating 2‑MAGs and NAEs. Long-term oil supplementation providing specific fatty acids (FA) was found to alter the levels of some NAEs and 2-AG^14,19^. Also, a positive association was found between the plasma composition of free FAs and some of their corresponding NAEs^20^. Moreover, gut microbiota composition has been associated with the endocannabinoidome in obesity-related dysbiosis or when altering bacterial populations with antibiotics and probiotics^21–26^. Gut bacterial communities and the host endocannabinoidome seem to be interrelated in a mutual crosstalk controlling whole body metabolism, but this association remains to be defined in humans^25,27^. Recent studies showed that some NAEs directly affect mice gut microbiota composition in vivo and in vitro whereas, in turn, germ-free mice exhibit significant alterations in the gut levels of these mediators^26,28^.

These findings support the hypothesis that, beyond body composition, dietary FA intake and gut microbiota, two potentially related factors, may determine the levels of NAEs and 2‑MAGs in humans^29^. Yet, very few data are available for most NAEs and 2‑MAGs and little is known on their correlations with body composition, the dietary intakes and gut microbiota composition. The present study aims at filling this knowledge gap by using a heterogeneous and overall healthy population examined either under free or controlled feeding conditions.

## Materials and methods

### Study cohorts

#### Cross-sectional sample

Subjects were recruited (NCT03463304) at the Institute of Nutrition and Functional Foods (INAF, Québec, Canada) and included 195 men and women (Table 1). Subjects with enteropathies, alcohol consumption exceeding the Canadian recommendation for men (> 15) and women (> 10 drinks/week), weight change (± 5 kg) in the last 6 months, having taken antibiotics in the last 3 months and pregnant and/or breastfeeding women were not eligible. Retrospectively, we notice that 25 participants declare to consume probiotics but only 15 consume probiotics on a regular basis considering very few reach the complete posology recommends by manufacturers.

**Table 1:** Anthropometric and metabolic characteristics of the subjects of the cross-sectional cohort.

	Men (n = 93)		Women (n = 102)		p value
	Mean (SD)	Range	Mean (SD)	Range	
Age (year)	42 (19)	20–85	40 ± 17	19–77	NS
BMI (kg/m^2^)	25.5 (4.2)	17.3–36.8	24.6 ± 5.0	13.3–42.0	NS
Overweight	38	–	23	–	–
Obesity	12	–	17	–	–
Waist circumference (cm)	91.4 (13.4)	70.0–130.8	83.0 (14.1)	60.0–121.0	< 0.001
Fat mass (kg)	20.2 (9.0)	7.2–43.3	23.6 (10.5)	4.7–54.0	0.02
Visceral adipose tissue (kg)	0.90 (0.91)	0.01–4.32	0.45 (0.56)	0–2.27	< 0.001
Fasting glucose (mmol/L)	5.1 (0.8)	3.7–10.2	4.8 (0.6)	3.9–8.7	< 0.001
HOMA-IR	1.6 (1.1)	0.2–5.9	1.4 (1.0)	0.3–6.0	NS
HbA1c (%)	5.3 (0.5)	4.3–8.2	5.2 (0.4)	4.4–7.3	NS
Triglycerides (mmol/L)	1.2 (0.6)	0.4–4.5	1.0 (0.5)	0.4–3.7	0.02
Total cholesterol (mmol/L)	4.5 (1.1)	1.8–7.7	4.7 (1.1)	2.2–7.6	NS
HDL cholesterol (mmol/L)	1.4 (0.4)	0.6–2.5	1.8 (0.4)	0.9–3.3	< 0.001
LDL cholesterol (mmol/L)	2.6 (0.9)	0.7–5.2	2.5 (0.9)	0.6–5.5	NS

Values are expressed as mean (SD) or n.

#### Controlled feeding intervention group

The group (NCT03783260) included 21 healthy men and women with a normal weight BMI recruited at INAF using the exclusion criteria described above. This was a fixed sequence isocaloric feeding study with a 13-day Control diet (i.e. low in fiber, rich in saturated fat) followed by a 2-day Mediterranean diet period (i.e. rich in oleic acid and in EPA and DHA). All foods and caloric beverages were provided to participants. Details about the composition of the Mediterranean diet are given in the Supplementary Table S2. Subjects were instructed to consume only the foods and beverages provided to them, which corresponded to their estimated energy needs. Energy needs for each subject were estimated by averaging the energy requirements estimated by a validated 3-day web-based 24 h dietary recall (R24W) and energy expenditure obtained with Harris–Benedict formula^30^. The Control diet was designed to reflect current Canadian macronutrient intakes and does not cause short-term nutritional deficiencies. The Mediterranean diet is characterized by greater intake of fruit and vegetables, plant-based proteins and whole grains^31^. It contains higher amount of monounsaturated FAs (MUFA), omega-3 PUFA and fibers, while less saturated FA (SFA) and simple sugar. Written informed consent was obtained and both projects were approved by the Laval University Ethics Committee (2017-328 and 2018-262).

### Dietary assessment

Participants of the cross-sectional sample were invited via e-mail to complete a web-based, self-administered 24 h dietary recall (R24W) on the day before the study visit and on two other unannounced days selected randomly during the week before the study visit using an in-house computer algorithm. Participants had 24 h to complete each recall. Details about the development and validation of the R24W have been reported elsewhere^32^.

### Body composition and sample collection

In the cross-sectional sample, body composition and fat distribution were assessed with a dual energy X-ray absorptiometry scanner (DXA, Lunar Prodigy Bone Densitometer, GE Healthcare Lunar, Madison, WI, USA) by trained professionals using the Lunar enCORE software version 14.1^33^. Overnight fasting blood samples were drawn at each study visit in both the cross-sectional and the controlled feeding intervention. The fecal collection was carried out the day prior to the study visit of the cross-sectional sample and immediately aliquoted and frozen by the participant.

### Circulating NAEs and 2‑MAGs

Levels of NAEs and 2‑MAGs in plasma samples (200 uL) were measured using high-performance liquid chromatography coupled to tandem mass spectrometry (LC–MS/MS) as previously described^34^. It allows the quantification of NAEs including AEA, *N*-palmitoyl‑ethanolamine (PEA), *N*-oleoyl‑ethanolamine (OEA), *N*-linoleoyl-ethanolamine (LEA), *N*-docosapentaenoyl‑ethanolamine (DPEA), *N*-eicosapentaenoyl-ethanolamine (EPEA) and *N*-docosahexaenoyl‑ethanolamine (DHEA), as well as 2-MAG including 2-AG, 2-palmitoyl-glycerol (2-PG), 2-oleoyl-glycerol (2‑OG), 2-linoleoyl-glycerol (2-LG), 2-eicosapenaenoyl-glycerol (2-EPG), 2-docosapentaenoyl‑glycerol (2-DPG) and 2­docosahexaenoyl-glycerol (2‑DHG). Monoacyl-glycerol isomers at positions 1 and 2 can be differentiated, but given their rapid interconversion, and the preferential esterification of PUFA on the *sn*-2 position of phospholipids, MUFA and PUFA-derived 2‑MAGs were summed and identified as 2-MAGs.

### 16S rRNA gene sequencing

Stool bacterial DNA was extracted using the QIAamp DNA Stool Kit (QIAGEN, CA, USA) and amplification of the V3–V4 region was performed using the primers 341F (5′-CCTACGGGNGGCWGCAG-3′) and 805R (5′-GACTACHVGGGTATCTAATCC-3′) (Illumina, CA, USA) as previously described^25^. Briefly, libraries were purified using magnetic beads (Axygen Biosciences, CA, USA) and quality assessed (Agilent Technologies, CA, USA). High-throughput sequencing (2 × 300 bp paired-end) was performed on a MiSeq. Sequences were processed using the Dada2 package (Version 1.10.1)^35^ and associations to bacterial taxa was obtained using the Silva v132 reference database^36^. Sequences present in fewer than 5 samples were filtered out and bacterial abundances were normalized using Cumulative Sum Scaling (CSS, MetagenomeSeq R package)^37^.

### Statistical analyses

Correlation coefficients (Pearson or Spearman) as well as partial correlation coefficients (ppcor package) were computed^38^. One-way analysis of variance (ANOVA) and Tukey HSD post hoc test were performed to compare parameters between tertiles or clusters. Unsupervised Hierarchical Clustering on Principal Components (*HPCP*, *FactoMineR* R package) was used to stratify subjects into clusters^39^. Paired t-tests were used to compare the control diet and the Mediterranean diet intervention. Partial Spearman’s rank correlations for gut bacteria taxa were computed using the PResiduals package^38^. Stepwise linear regression analysis was employed to identify families that best predicted individual NAEs and 2-MAGs in models including adiposity measures and self-reported dietary FA intakes (jtools package). Values of NAEs and 2‑MAGs below the limit of quantitation or beyond two SD from the mean were considered as outliers. All statistical analyses were conducted with R software version 3.4.3.

### Ethical approval

All experiments and methods were performed in accordance with relevant guidelines and regulations.

## Results

NAEs and 2‑MAGs derived from saturated (i.e. palmitic), monounsaturated (i.e. oleic) and polyunsaturated omega-6-FA (i.e. arachidonic, linoleic) and polyunsaturated omega-3-FA (i.e. EPA, DPA and DHA) were identified in the plasma of almost all participants of the cohort (Tables 1, 2). Circulating NAEs were less abundant than their corresponding 2‑MAG congeners (Table 2). We observed significant differences between men and women for almost all 2-MAGs, but not for NAEs. We tested for sex difference in a subset of men (n = 59) and women (n = 59) matched for age and BMI. No sex difference was found in circulating NAE and 2‑MAG levels, with the exception of slightly higher 2-DPG levels in women (Table 2).

**Table 2:** Circulating NAEs and 2‑MAGs in the entire cohort and in a subset of men and women matched for age and BMI.

Mediators (pmol/ml)	Entire cohort (n = 195)	Matched for age and BMI		p value
		Men (n = 59)	Women (n = 59)	
***N*-acylethanolamines (NAEs)**				
AEA	0.89 ± 0.32	0.89 ± 0.33	0.94 ± 0.30	NS
PEA	6.93 ± 3.69	7.55 ± 5.54	6.42 ± 1.75	NS
OEA	5.60 ± 1.91	6.08 ± 2.20	5.59 ± 1.69	NS
LEA	1.91 ± 0.90	2.04 ± 1.17	1.90 ± 0.85	NS
EPEA	0.07 ± 0.05	0.08 ± 0.06	0.07 ± 0.06	NS
DHEA	1.08 ± 0.44	1.17 ± 0.51	1.07 ± 0.49	NS
**2-monoacylglycerols (2-MAGs)**				
2-AG	6.45 ± 4.64	6.32 ± 5.63	6.39 ± 3.64	NS
2-PG	10.4 ± 7.81	10.6 ± 10.4	10.4 ± 6.04	NS
2-OG	43.1 ± 32.2	42.3 ± 46.4	43.0 ± 23.5	NS
2-LG	66.3 ± 39.4	62.8 ± 47.8	65.1 ± 33.2	NS
2-EPG	1.37 ± 1.35	1.40 ± 1.51	1.50 ± 1.55	NS
2-DPG	3.07 ± 2.42	2.10 ± 1.64	3.39 ± 2.13	< 0.001
2-DHG	3.71 ± 3.32	3.81 ± 3.74	3.97 ± 3.72	NS

Values are expressed as mean ± SD.

### Body composition

Table 3 shows associations between circulating levels of NAEs and 2‑MAGs and adiposity measures. Except for OEA, all other mediators were positively correlated with total and visceral fat masses. Similarly, these mediators were correlated with BMI (0.14 < r < 0.37; p < 0.05) and waist circumference (0.17 < r < 0.47; p < 0.05). Associations between circulating NAE levels and visceral fat mass were lost after adjustment for total fat mass. In contrast, levels of all 2‑MAGs remained significantly associated with visceral adiposity after adjustment for fat mass.

**Table 3:** Pearson correlation coefficients between circulating NAEs and 2‑MAGs and body composition and fat distribution.

	Fat mass (kg)	Visceral fat mass (kg)	
		Unadjusted	Adjusted for fat mass
***N*-acylethanolamines (NAEs)**			
AEA	0.35**	0.33**	0.12
PEA	0.39**	0.35**	0.12
OEA	0.12	0.16*	0.11
LEA	0.23*	0.20*	0.05
EPEA	0.45**	0.38**	0.11
DHEA	0.24**	0.20*	0.05
**2-monoacylglycerols (2-MAGs)**			
2-AG	0.35**	0.47**	0.34**
2-PG	0.25**	0.36**	0.27**
2-OG	0.26**	0.46**	0.39**
2-LG	0.13	0.32**	0.32**
2-EPG	0.33**	0.54**	0.45**
2-DPG	0.20*	0.44**	0.42**
2-DHG	0.33**	0.41**	0.26**

*p-value < 0.01, **p-value < 0.001.

**Figure 1:**
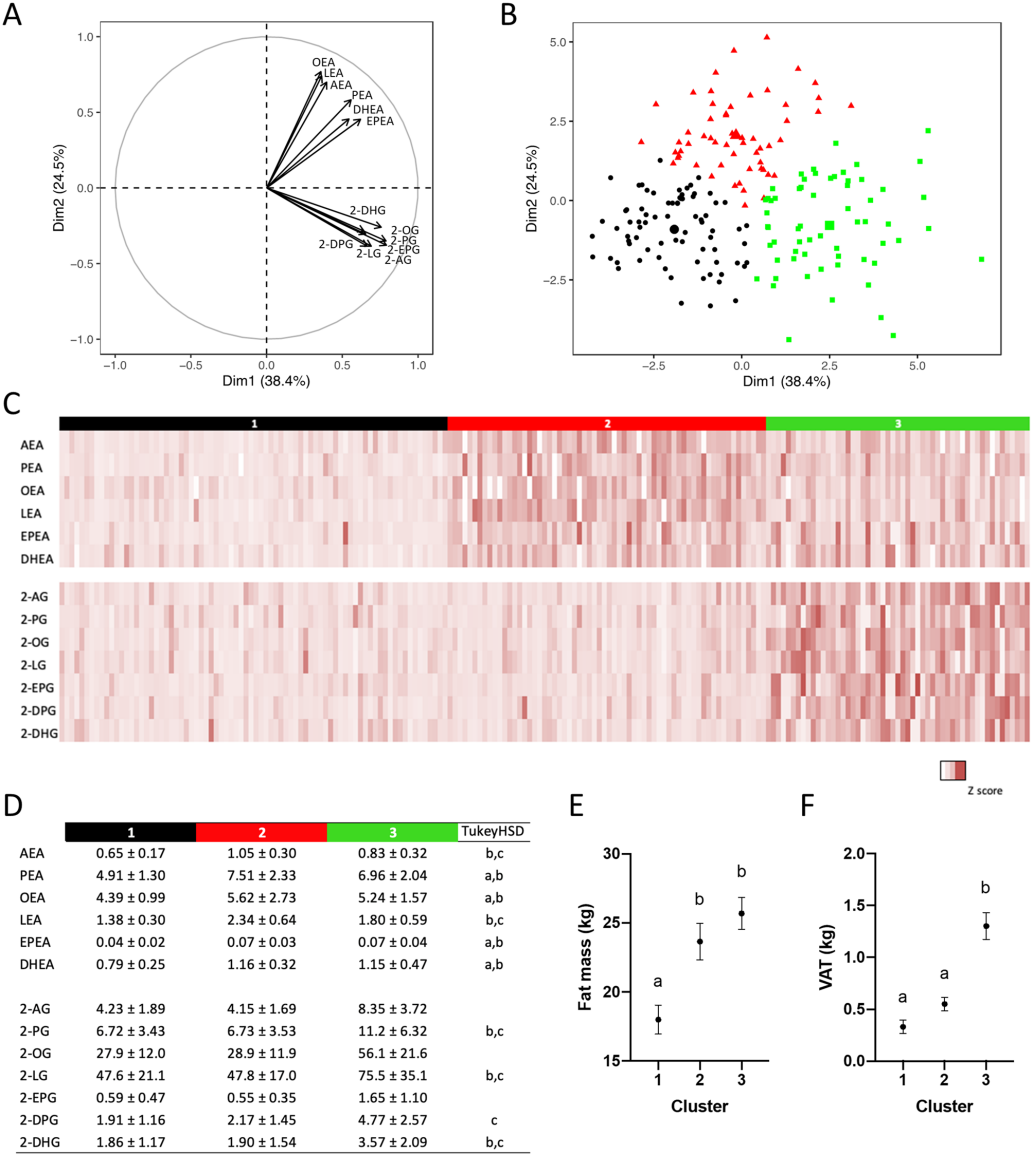
Hierarchical clustering based on principal component analysis (PCA) of circulating NAEs and 2‑MAGs (n = 195). (**A**) Loading plot of the principal component analysis including all circulating levels of NAEs and 2-MAGs. (**B**) PCA visualization of NAE and 2‑MAG profile for each individual. Colors indicate the 3 clusters determined using hierarchical clustering. (**C**) Heatmap representation of normalized levels of each NAE and 2‑MAG according to the clusters. (**D**) Mean ± SD of circulating NAEs and 2‑MAGs by clusters. Analysis of variance and Tukey HSD post hoc statistics are shown: (a) 1 vs 2; (b) 1 vs 3 and (c) 2 vs 3. (**E**) Fat mass (kg) and (**F**) visceral adipose tissue mass (kg) according to clusters with analysis of variance and Tukey post hoc test. Letters indicate significant differences between clusters (p < 0.05).

Both NAEs and 2‑MAGs appear to distinctly drive separation of subjects as shown in the factor loading plot (Fig. 1A). This analysis reveals that the levels of the congeners are highly interrelated within each family. Unsupervised hierarchical clustering of subjects based on their circulating NAE and 2‑MAG levels has led to three subgroups with distinctive profiles (Fig. 1). Subjects from cluster 1 (n = 80) had generally low circulating levels of all mediators, while subjects from cluster 2 (n = 66) and cluster 3 (n = 57) had respectively elevated levels of NAEs and 2-MAGs (Fig. 1C). In addition, subjects from the three clusters were also characterized by different body composition and fat distribution profiles. Subjects from clusters 2 and 3 had higher fat mass than those from cluster 1, while only subjects from the cluster 3 had significantly elevated visceral fat mass compared with subjects from cluster 1.

### Self-reported dietary intakes

**Figure 2:**
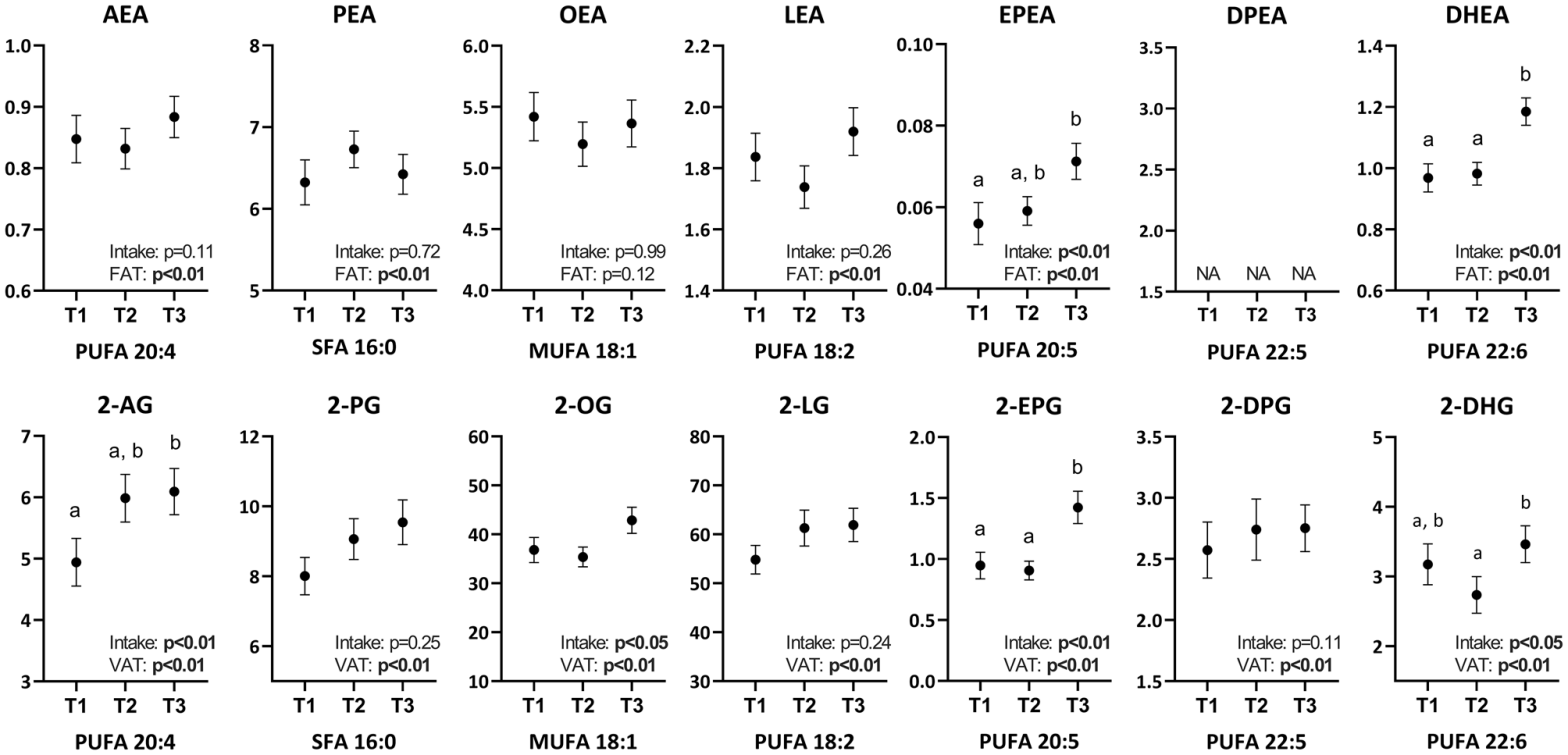
Circulating NAE and 2‑MAG levels stratified by tertiles of self-reported fatty acid intakes. Values are mean ± SEM. Letters indicate significant differences between tertiles in analysis of variance and Tukey post hoc test (p < 0.05; n = 47 to 71 per tertile). Results of the generalized linear model including the mediators as the dependent variables and fat mass (NAEs) or visceral fat mass (2‑MAGs) as independent variables are included in each graph.

We next investigated if self-reported dietary intakes correlate with the circulating levels of NAEs and 2‑MAGs. Only few relatively weak associations were noted with macronutrient intakes regardless of adjustment for total or visceral fat mass (Supplementary Table S1). However, we observed higher levels of omega-3-FA-derived NAEs and 2‑MAGs (i.e. EPEA, 2-EPG, DHEA and 2-DHG) in subjects with higher self-reported intakes of EPA or DHA respectively (Fig. 2). In addition, circulating levels of 2-AG were higher in the subjects with a high self-reported intake of arachidonic acid. These associations remained significant after adjustment for total or visceral adiposity (Fig. 2).

### Gut microbiota taxa

**Figure 3:**
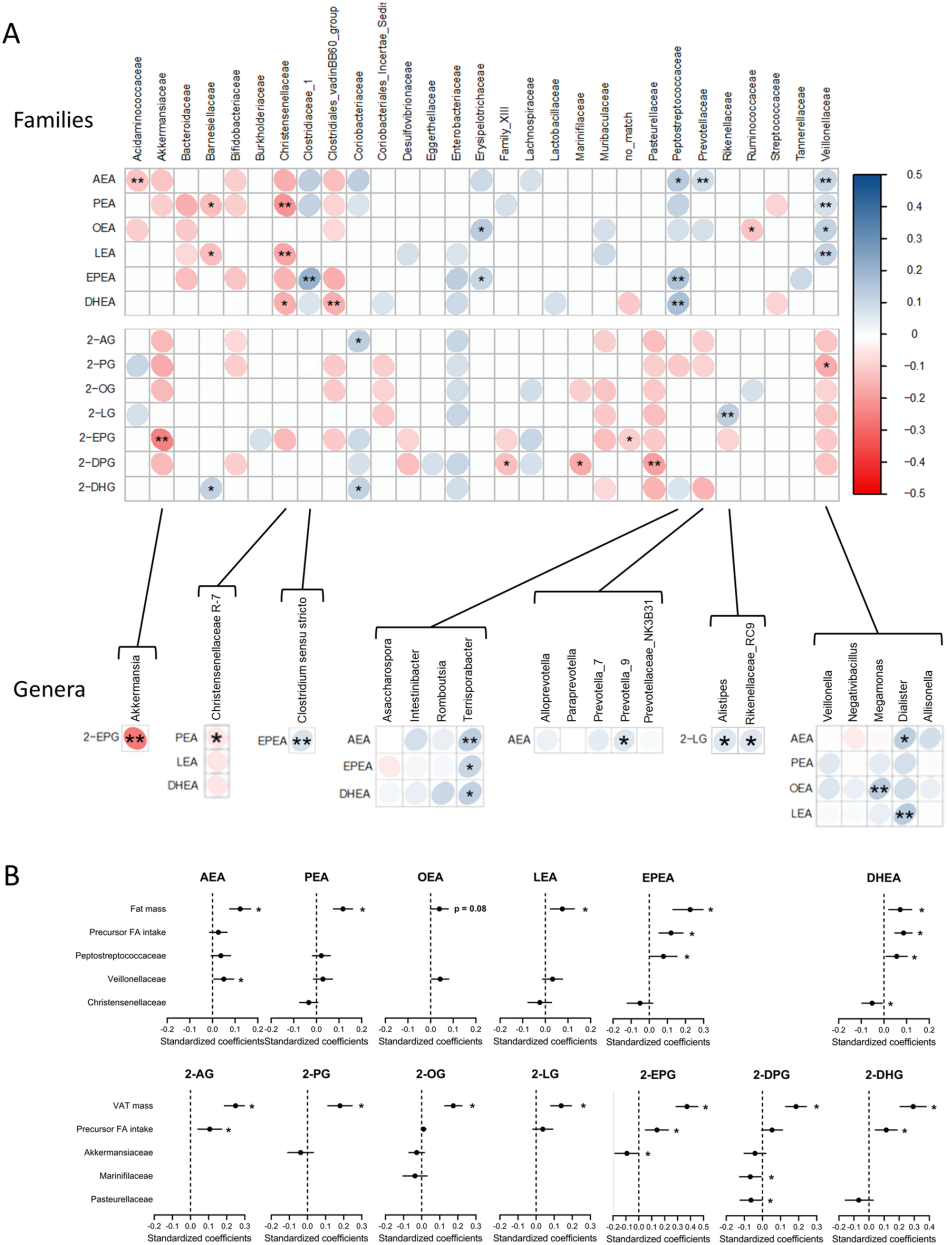
Circulating NAE and 2‑MAG levels association with gut bacterial taxa independent of adiposity measures. (**A**) Heatmap of Spearman's rank correlation coefficients of the relative abundances of gut microbiota taxa and the levels of circulating NAEs and 2‑MAGs. The analysis includes all gut microbiota families (Top) as well as the genera from families significantly associated with the mediators (Bottom). Circle color intensity represents the magnitude of the unadjusted correlation (Spearman rho coefficients). Blue circles indicate positive correlations and red circles negative correlations. Significant correlations following adjustment for total fat mass (NAEs) or visceral fat mass (2‑MAGs) are shown (*p < 0.1, **p < 0.05; n = 195). (**B**) Standardized regression coefficients of independent predictors of circulating NAE and 2-MAG levels. Results of the generalized linear model including the mediators as the dependent variables and fat mass (NAEs) or visceral fat mass (2‑MAGs) as independent variables are included in each graph. All bacterial families significantly associated to the mediator were considered in this analysis. All models also include the total or visceral fat mass as well as the dietary intake of the fatty acid precursor. Stepwise selection procedure was used to compute final models (*p < 0.05).

In view of the recent link between the gut microbiota, adiposity and the endocannabinoidome, we investigated if circulating NAE and 2-MAG levels depend on gut microbiota taxa and if these putative associations remain independent of adiposity and dietary intakes. We first correlated individual circulating NAE and 2-MAG levels with bacterial family relative abundances (Fig. 3A, Top). As endocannabinoidome and gut microbiota are both correlates of adiposity measures, these associations were further adjusted for total or visceral fat mass, separately for NAEs and 2‑MAGs respectively. We mainly observed that the relative abundance of *Christensenellaceae* was negatively associated, while those of *Peptostreptoccocaceae* and *Veillonellaceae* families were positively associated, with several NAEs independently of adiposity measures. In contrast, circulating 2-MAG levels showed only a few independent associations with the relative abundance of bacterial families. Notably, *Akkermansiaceae* was negatively associated with 2-MAG, especially 2-EPG, levels. We also observed that within these families, one or more genera were generally associated with the same NAE and 2-MAG congeners (Fig. 3A, Bottom). Linear regression models including all putative contributors of circulating NAE and 2-MAG levels confirmed that these bacterial families combined with dietary intakes of the corresponding FAs and adiposity measures were, to different extents, independent predictors of circulating NAE and 2-MAG congeners (Fig. 3B).

### Short-term dietary intervention

**Figure 4:**
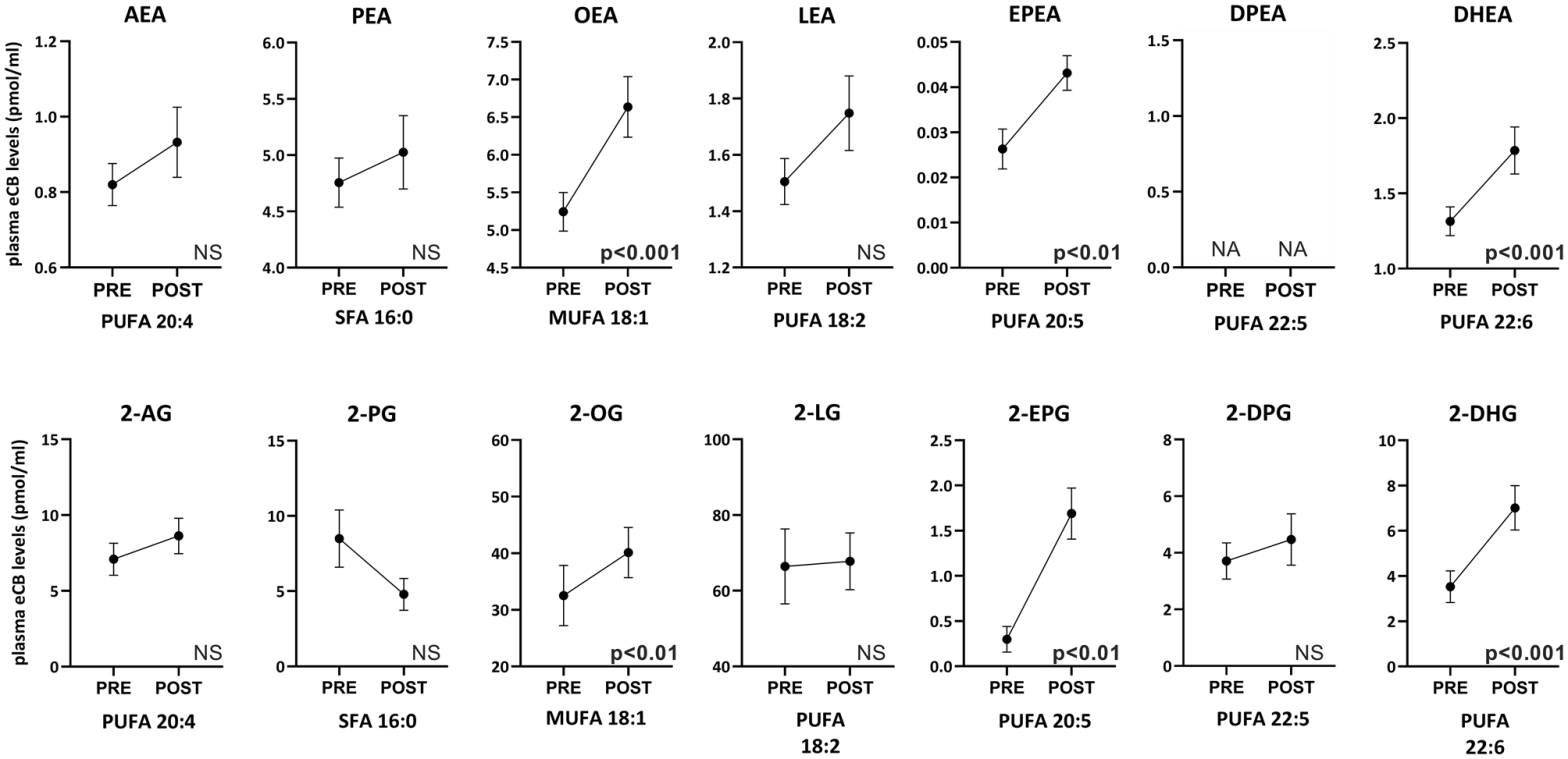
Circulating NAE and 2‑MAG levels following the 13-day control diet (PRE) and after the 2 days on the Mediterranean diet (POST). Values are mean ± SEM. n = 21 except for EPEA (n = 19), 2-EPG (n = 9), 2-PG (n = 9) and 2-DPG (n = 20). Paired t-test between PRE and POST dietary intervention (p < 0.05). *ND* not determine.

We next assessed whether the circulating levels of NAEs and 2-MAGs were impacted by a short-term isocaloric dietary intervention reflective of the Mediterranean diet. Noteworthy, a 2‑day Mediterranean diet period was sufficient to increase the levels of most omega‑3-FA-derived endocannabinoidome mediators (i.e. EPEA, DHEA, 2‑EPG and 2‑DHG) compared with values measured after the 13-day control diet (Fig. 4). Similarly, NAEs and 2‑MAGs derived from oleic acid (i.e. OEA and 2-OG) were increased after 2 days on the Mediterranean diet. These results are consistent with higher intakes of oleic acid as well as of EPA and DHA during the Mediterranean diet compared to the control diet. In contrast, linoleic, arachidonic and palmitic acids-derived NAEs and 2-MAGs were not modified by the Mediterranean diet although the dietary intakes of linoleic were increased, and those of arachidonic and palmitic acids were reduced compared with the control diet, suggesting that transformation of linoleic acid into arachidonic acid may have compensated the change in dietary FA intakes.

## Discussion

This study was designed to investigate the influence of sex, body composition, fat distribution, dietary habits and gut microbiota on the circulating levels of NAEs and 2-MAGs, which are part of the endocannabinoidome. Previous studies reported such associations for the circulating endocannabinoids (i.e. AEA and 2‑AG) in individuals with obesity, overweight, prediabetes or type 2 diabetes. With this study, we investigated for the first time all most abundant endocannabinoid congeners and targeted a relatively large sample of overall healthy subjects under conditions of either self-reported intake or short-term dietary intervention. The endocannabinoidome-related mediators investigated—the NAEs (AEA congeners) and the 2‑MAGs (2-AG congeners)—share the same metabolic pathways with endocannabinoids but act on additional/different receptors [for review, see^40^]. We report that body composition and fat distribution are strongly associated to the circulating levels of almost all NAEs and 2-MAGs. However, specific patterns were observed in each family—NAEs being associated with the total fat mass and 2-MAGs mainly driven by specific fat accumulation in the visceral compartments. Independent of the total or visceral fat mass, several NAE and 2-MAG congeners were associated with self-reported intake of the corresponding FA precursors and modified by a short-term dietary intervention. Moreover, the relative abundance of some bacterial families, such as *Veillonellaceae*, *Peptostreptococcaceae* and *Akkermansiaceae*, were independently correlated with the blood levels of several members of the NAE or 2-MAG families of lipids.

Adiposity measures have been previously reported as strong correlates of circulating levels of endocannabinoids^13,17,18^. We corroborate these findings and we further demonstrate that their congeners, except OEA, are also correlated with body composition and fat distribution. Interestingly, we revealed that NAEs and 2-MAGs are differently associated with these variables. Individuals characterized by subcutaneous adipose tissue accumulation have greater levels of NAEs compared to lean individuals, while those characterized by visceral adipose tissue accumulation have greater levels of both NAEs and 2‑MAGs. Previous studies have observed higher circulating levels of 2-AG, but not of AEA, in individuals with high intra-abdominal adiposity compared to lean or obese individuals with low intra-abdominal adiposity^17,18^. Our results strengthen the link between circulating 2-AG levels and visceral adipose tissue and extend this knowledge to all 2-AG congeners.

We could posit that production of NAEs and 2-MAGs by specific adipose tissue compartment contribute to their circulating levels, but this remains to be demonstrated. Indeed, more studies are needed to infer the direction of causality, if any, of total or visceral fat mass and circulating levels of NAEs and 2‑MAGs. Intriguingly, we found that total and visceral fat mass are associated with elevated levels of lipid mediators known to activate a wide range of receptors that are associated with likely detrimental (i.e. CB_1_) and beneficial (e.g. GPR55, GPR119, PPARs, TRPV1) actions in the context of metabolic diseases. The lack of detailed affinity and specificity data for each mediator, especially omega-3-FA-derived NAEs and 2-MAGs, for these receptors, and the paucity of information regarding the exact role of some of these proteins in the context of adipocyte biology, do not allow to draw a definitive portrait of endocannabinoidome tone in relation to adipose tissue accumulation and distribution. We speculate that endocannabinoidome mediators modulate fat accumulation and distribution via their actions on food intake, lipogenesis, insulin sensitivity, among others. Conversely, excess accumulation or distribution of fat may lead to altered circulating levels of NAEs and 2‑MAGs as either adaptive or maladaptive responses.

Interestingly, the association between NAEs and adiposity measures was not observed for OEA. Côté et al. also report such a discordant relationship between AEA and OEA^18^. Moreover, experiments in rodents showed that small intestine production of OEA is disrupted in the gut following diet-induced obesity^41^. Despite being closely related to AEA, OEA is not a ligand of CB_1_ and CB_2_ receptors^2^. OEA is involved in the regulation of satiety signals by fat sensing in the gut via PPARα signaling^42^, but also promotes fat oxidation, incretin release and reduces upper gut motility via PPARα and GPR119-dependent and -independent mechanisms^43^. The key and specific role of OEA as a fat sensor in the proximal small intestine and, subsequently, as an inhibitor of the intake of fat may necessitate that the levels of this mediator are independent from the pre-existing amounts of body fat, and hence may explain why they are not associated with adiposity measures, unlike other NAEs.

The lack of a clear association between self-reported macronutrient intake and the profile of endocannabinoid-related effectors is rather surprising considering their link with metabolic variables and their alleged role at the crossroad of the environment and the host energy homeostasis^44^. Nevertheless, we observed interesting effects when investigating individual FA species in both the cross-sectional and dietary intervention studies. We revealed that omega-3-PUFA-derived NAEs and 2‑MAGs were responsive to the dietary intake of their corresponding FA precursors. It was not surprising to find slightly different associations as these differences likely reflect the fact that NAEs and 2‑MAGs are ultimately derived from FAs differentially sensitive to different dietary FAs. Our data may lead to the speculation that dietary omega-3-PUFAs ultimately become amidated to the ethanolamine moiety of phosphatidylethanolamine more than other FAs, thereby yielding to *N*‑acylphosphatidylethanolamine and serving as a source of NAEs^2^.

Regimen-induced increase of oleic acid and omega-3 FAs, such as with a Mediterranean diet, resulted in significant and similar increases in both the NAEs and 2-MAGs derived from these FAs. The levels of 2-AG, although positively associated with increased self-reported intake of arachidonic acid, remained stable during the dietary intervention poorer in this FA. This lack of sensitivity to acutely reduce arachidonic acid intake may arise from elongation/desaturation of linoleic acid to arachidonic acid prior to its incorporation into phospholipids and, ultimately, NAEs and 2-MAGs. Accordingly, 2‑LG and LEA levels did not increase following the Mediterranean diet.

Noteworthy, we demonstrate that these relations with intakes of specific FA precursors were independent of adiposity measures. In fact, other studies had shown that omega-3-FA supplementation decreases circulating AEA and 2‑AG levels and increases omega-3-derived congeners (i.e. EPEA)^14,19,45,46^, while MUFA supplementation promotes higher circulating OEA levels^19^. Joosten et al. also showed that AEA, OEA and PEA were correlated to circulating levels of the corresponding FA precursors^20^. However, the role of adiposity and fat distribution in these effects was not investigated. Our study clearly indicates that the profile of dietary FAs is a key independent determinant of the wider endocannabinoidome in circulation, not only using self-reported dietary intake data, but also following a short-term dietary intervention.

Since recent studies showed that NAEs in the small intestine are elevated in germ-free mice, and that a cocktail of AEA, PEA, OEA and LEA promotes in vitro growth of some mouse gut bacteria taxa, including those belonging to the family *Veillonellaceae*, we have investigated here whether gut microbiota are associated with these mediators^28^. We found that, indeed, increased relative abundance of some bacterial families, including not only *Veillonellaceae*, but also *Christensenellaceae*, *Peptostreptococcaceae* and *Akkermansiaceae*, can be associated with circulating endocannabinoidome mediators, independently of adiposity and dietary FA intakes. Bacteria in the *Veillonellaceae* family, which were correlated positively with non-omega-3 PUFA-derived NAEs, produce short-chain FAs (SCFA), especially propionate, associated with metabolic health^47^. Interestingly, *Veillonellaceae* genera seem differently associated with host metabolism: whilst the relative abundance of *Phascolarctobacterium* at baseline was negatively associated with adiposity, the opposite was found for the *Dialister* and *Megamonas* genera^48^. In the present study, we found that two genera from the *Veillonellaceae* family, *Megamonas* and *Dialister*, were associated to NAEs. It was suggested that members of the *Veillonellaceae* family have varying impact on energy metabolism, and, in view the different roles of AEA, OEA and LEA in this context, our results reinforce this hypothesis. *Christensenellaceae* is one of the most consistently associated families with adiposity^49^. Indeed, the relative abundance of *Christensenellaceae* was inversely related to host BMI in different populations and to the presence of several diseases, including obesity and inflammatory bowel disease^50–52^. Very little data are available for the *Peptostreptococcaceae* family*,* as well as for its *Romboutsia* and the *Terrisporobacter* genera, and their link with metabolism. A potential link reported between PUFA intake and this group of diverse anaerobes with fermentative type of metabolism^29^, may support its association, observed here, with omega-3 PUFA-derived NAEs. Finally, strong evidence supports the role of *Akkermansiaceae*, especially *A. muciniphila*, in metabolic health^53^. The present finding of a negative association of *Akkermansiaceae* with circulating 2‑EPG, and of *Marinifilaceae* and *Pasteurellaceae* with circulating 2-DPG, two poorly studied 2-AG congeners, remains to be clarified. In summary, we show that dietary FA intakes and adiposity are not the only determinants of circulating endocannabinoidome mediators, and that NAEs are associated with different families of bacteria in the human gut microbiota, in agreement with previous mechanistic studies in rodents.

The fact that both self-reported FA intakes and very short term consumption of a Mediterranean diet rich in omega-3 PUFA and oleic acid can determine the profile of circulating endocannabinoidome mediators, independently of adiposity measures, is a key finding of this study and supports the concept that nutritional approaches may rapidly and profoundly affect the levels of these bioactive lipids. We also highlight the importance of carefully considering total and visceral adiposity in the design of studies aimed at measuring circulating NAEs and 2‑MAGs. More importantly, controlled feeding studies or a precise assessment of self-reported FAs dietary intake should be carried out before blood or tissue sampling for NAEs and 2‑MAGs profiling. Altogether, the results obtained here justify and may help designing future dietary interventions aimed at manipulating endocannabinoidome signaling to exploit the capability of various endocannabinoid congeners in producing potential beneficial effects on obesity and its comorbidities^43,45,54^. Finally, following our finding that some NAEs and 2‑MAGs are also associated with gut microbiota composition, interventions aimed at modulating the levels of these mediators might also find application in counteracting the metabolic disturbances linked to gut dysbiosis^26,27,55,56^.

## Supplementary information

Supplementary Information

## Data Availability

Raw sequence data have been submitted to NCBI (BioProject ID PRJNA644138 and SRA accession number SUB7687442). Individual de-identified subject data, including a data dictionary, related to the analyses included in this manuscript will be made available from the corresponding author on reasonable request (alain.veilleux@fsaa.ulaval.ca).
