# Supplementary material for: Dietary fatty acid intake and gut microbiota determine circulating endocannabinoidome signaling beyond the effect of body fat
Source: Sci Rep. 2020 Sep 29;10:15975. doi: 10.1038/s41598-020-72861-3 (PMC7524791; doi:10.1038/s41598-020-72861-3)
Supplement: Supplementary file 1 — Supplementary Information [file 41598_2020_72861_MOESM1_ESM.docx]

Dietary fatty acid intake and gut microbiota determine circulating endocannabinoidome signaling beyond the effect of body fat

Sophie Castonguay-Paradis^1,3,6^, Sébastien Lacroix^1,6^, Gabrielle Rochefort^1,3,6^, Lydiane Parent^3,6^, Julie Perron^1,6^, Cyril Martin^2,6^, Benoît Lamarche^1,3^, Frédéric Raymond^1,3,6^, Nicolas Flamand^2,4,6^, Vincenzo Di Marzo^1,2,3,4,5,6^, Alain Veilleux^1,3,6^*

**Affiliations:**

1. Centre Nutrition, Santé et Société (NUTRISS), Institut sur la nutrition et les aliments fonctionnels (INAF), Québec, Canada.

2. Centre de recherche de l’Institut universitaire de cardiologie et de pneumologie de Québec (IUCPQ), Québec, Canada.

3. École de nutrition, Faculté des sciences de l’agriculture et de l’alimentation (FSAA), Université Laval, Québec, Canada.

4. Département de médecine, Faculté de Médecine, Université Laval, Québec, Canada.

5. Joint International Unit on Chemical and Biomolecular Research on the Microbiome and its Impact on Metabolic Health and Nutrition (UMI-MicroMeNu).

6. Canada Research Excellence Chair in the Microbiome-Endocannabinoidome mediators Axis in Metabolic Health (CERC-MEND).

***Corresponding author:**

Alain Veilleux, PhD

Institut sur la nutrition et les aliments fonctionnels (INAF), Université Laval

2440, boulevard Hochelaga, Québec, Québec (Canada), G1V 0A6

Email: alain.veilleux@fsaa.ulaval.ca

Telephone : 418.656.2131 p.405108

**Supplementary Table 1.** Pearson correlation coefficients between circulating NAEs, 2-MAGs and dietary nutrient intakes adjusted for adiposity measures.

|  | **N-acyl-ethanolamines (NAEs)^#^** | | | | | |  | **2-monoacyl-glycerols (2-MAGs) ^#^** | | | | | | |
| --- | --- | --- | --- | --- | --- | --- | --- | --- | --- | --- | --- | --- | --- | --- |
|  | **AEA** | **PEA** | **OEA** | **LEA** | **EPEA** | **DHEA** |  | **2-AG** | **2-PG** | **2-OG** | **2-LG** | **2-EPG** | **2-DPG** | **2-DHG** |
| **Energy (kcal)** | -0.11 | -0.02 | -0.08 | -0.09 | -0.11 | -0.06 |  | 0.08 | 0.02 | 0.08 | 0.10 | 0.08 | **0.17*** | 0.05 |
| **Carbohydrate (g)** | -0.07 | -0.09 | -0.13 | -0.10 | **-0.15*** | -0.06 |  | 0.04 | 0.01 | 0.04 | 0.10 | 0.10 | **0.17*** | 0.04 |
| **Protein (g)** | -0.03 | -0.04 | **-0.15*** | -0.10 | -0.09 | -0.09 |  | 0.14 | 0.01 | 0.07 | 0.09 | 0.08 | **0.16*** | 0.08 |
| **Fat (g)** | -0.02 | 0.02 | -0.05 | -0.07 | -0.06 | -0.01 |  | 0.08 | 0.01 | 0.08 | 0.06 | 0.03 | 0.11 | 0.03 |
| **MUFA (g)** | 0.05 | 0.04 | -0.03 | -0.02 | -0.01 | 0.03 |  | 0.10 | 0.02 | 0.11 | 0.09 | 0.01 | 0.11 | 0.04 |
| **PUFA (g)** | 0.05 | 0.05 | -0.01 | 0.07 | -0.02 | -0.01 |  | 0.07 | -0.02 | 0.08 | 0.13 | 0.06 | 0.13 | 0.01 |
| **SFA (g)** | 0.01 | -0.01 | -0.06 | **-0.16*** | -0.09 | -0.03 |  | 0.06 | 0.10 | 0.05 | -0.02 | 0.04 | 0.09 | 0.04 |

*p<0.05. ^#^Correlations with NAEs were adjusted for total fat mass and those with 2-MAGs were adjusted for visceral fat mass.

**Supplementary Table 2:** Dietary composition of the Control and the Mediterranean diets.

| Nutrients | Control diet | Mediterranean diet |
| --- | --- | --- |
| Energy (kcal) | 2500 | 2500 |
| Carbohydrates (%E) | 47.2 | 47.2 |
| Proteins (%E) | 17.7 | 15.2 |
| Lipids (%E) | 35.1 | 35.1 |
| Alcohol (%E) | 0 | 2.5 |
| SFA (%E) | 15.3 | 6.0 |
| MUFA (%E) | 11.7 | 20.2 |
| PUFA (%E) | 5.01 | 6.7 |
| Arachidonic acid (g) | 0.23 | 0.13 |
| Palmitic acid (g) | 20.9 | 11.8 |
| Oleic acid (g) | 30.2 | 37.1 |
| Linoleic acid (g) | 12.8 | 16.5 |
| Eicosapentaenoic acid [EPA] (g) | 0.009 | 0.27 |
| Docosapentaenoic acid [DPA] (g) | 0.02 | 0.07 |
| Docosahexaenoic acid [DHA] (g) | 0.04 | 0.64 |

All nutrients are reported for an energy intake of 2500 kcal per day.
